# Supplementary figures and images for: Population genetic structure and hybrid zone analyses for species delimitation in the Japanese toad (Bufo japonicus)
Source: PeerJ. 2023 Oct 24;11:e16302. doi: 10.7717/peerj.16302 (PMC10607272; doi:10.7717/peerj.16302)

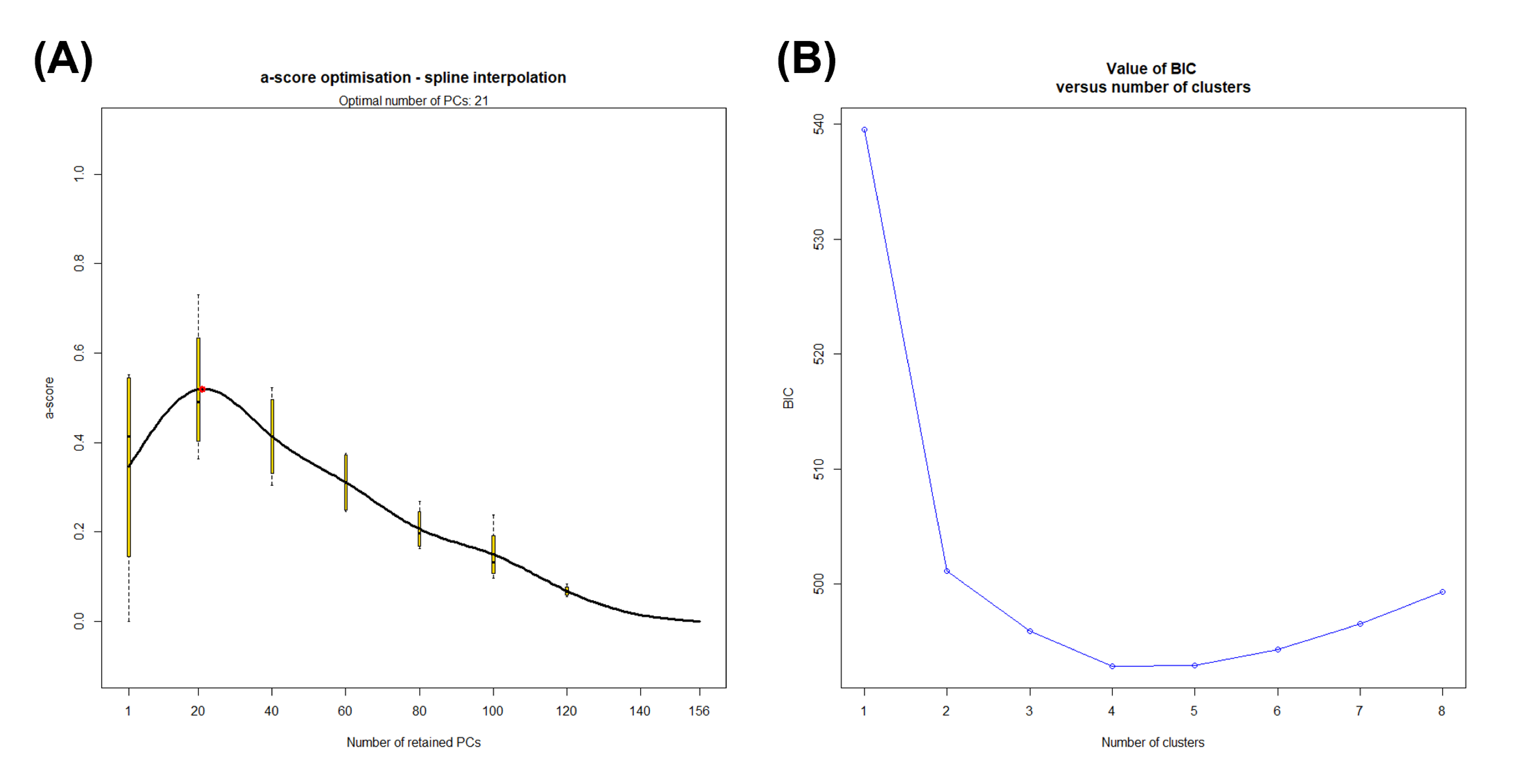

Supplement: Supplemental Information 1 [file peerj-11-16302-s001.png]

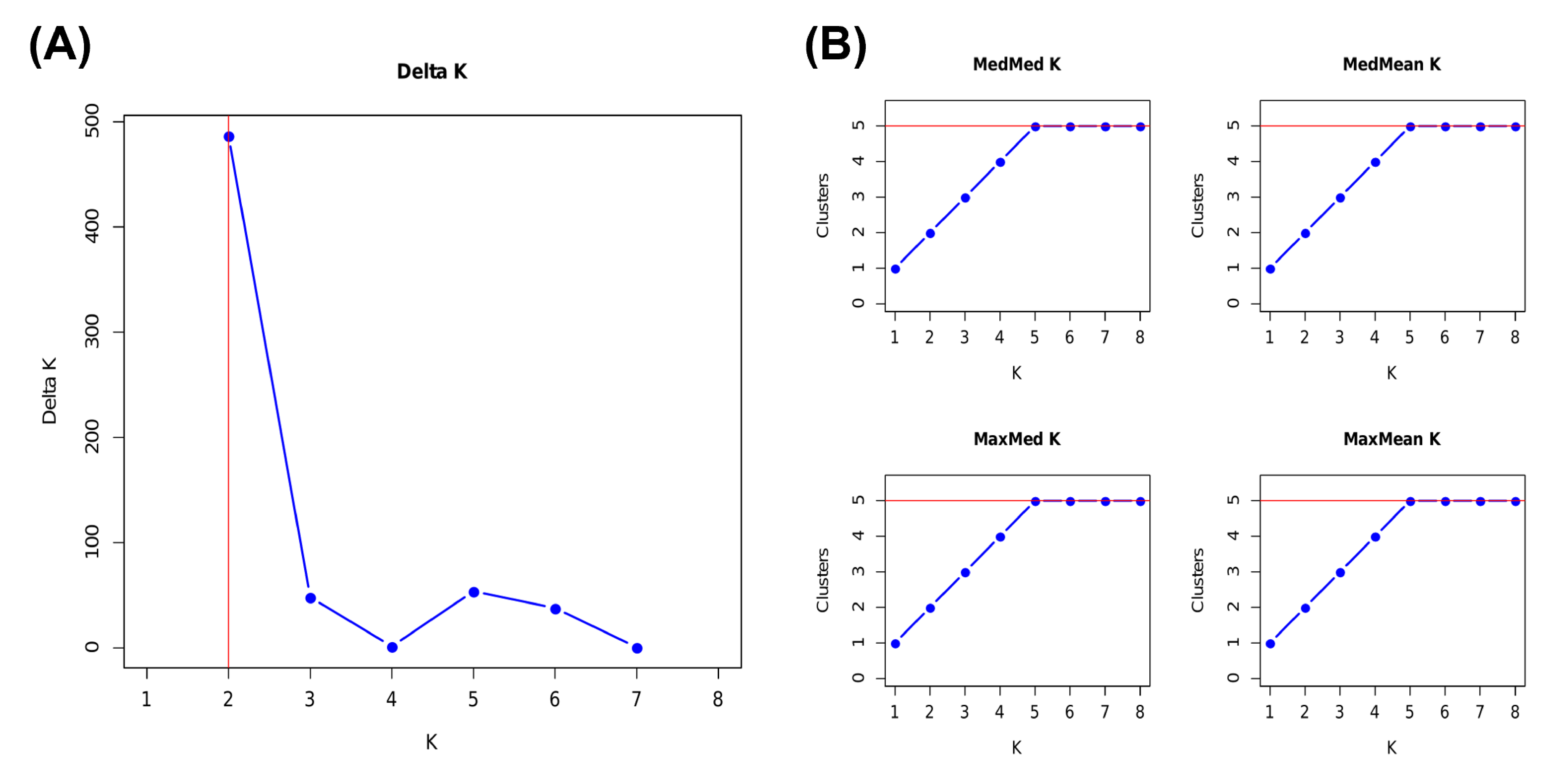

Supplement: Supplemental Information 2 [file peerj-11-16302-s002.png]

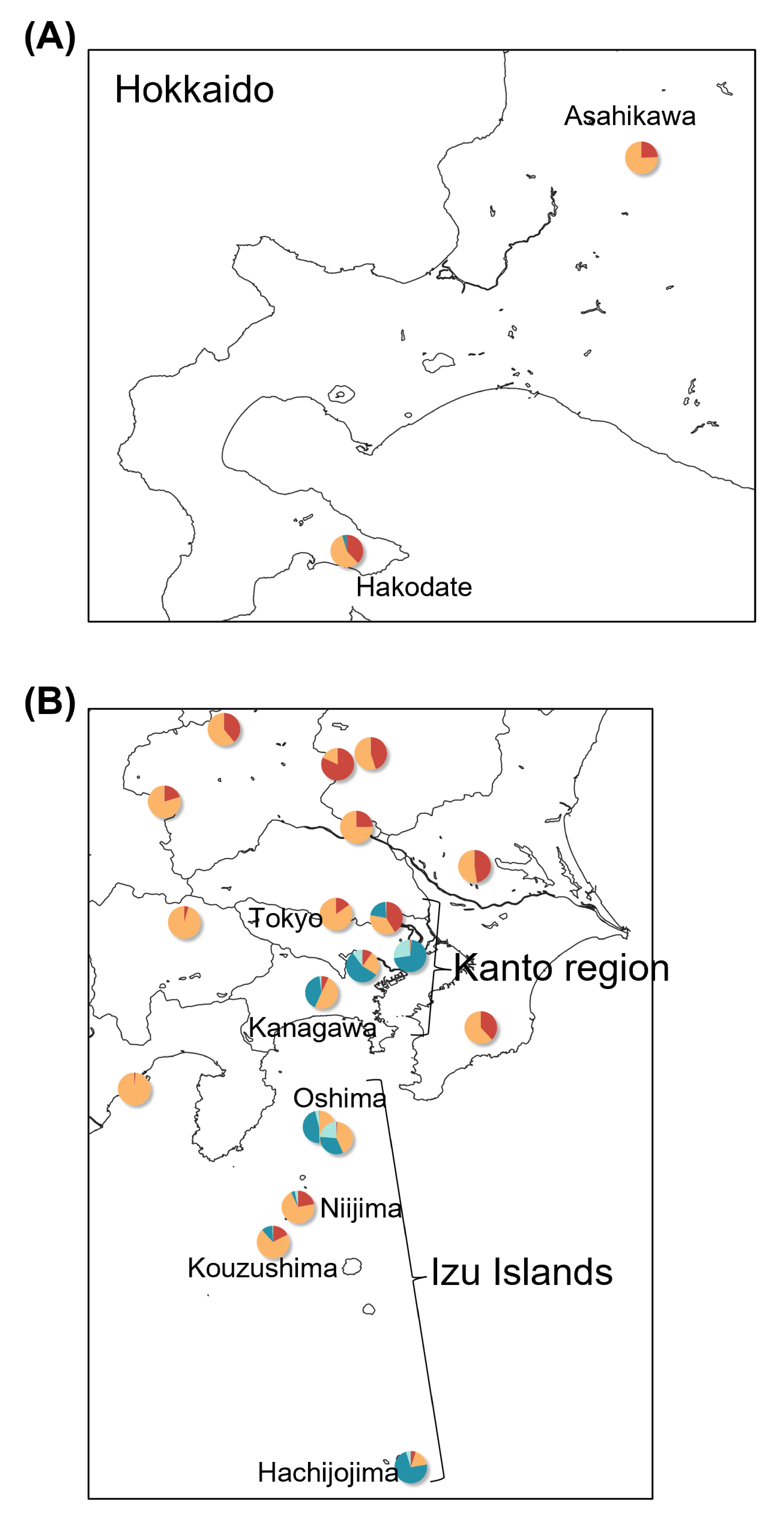

Supplement: Supplemental Information 3 — Pie charts show the q-values inferred by the Structure program for each individual. Color assignments for clusters were the same as in Fig. 4. [file peerj-11-16302-s003.png]
